# Environmental tobacco smoke and stress as risk factors for miscarriage and preterm births

**DOI:** 10.1007/s00404-012-2417-0

**Published:** 2012-06-21

**Authors:** Farha Arffin, Fouad H. AL-Bayaty, Jamiyah Hassan

**Affiliations:** 1 Faculty of Dentistry, Centre of Studies for Periodontology, Universiti Teknologi MARA, Level 19, Tower 2, Science and Technology Complex, 40450 Shah Alam, Selangor Malaysia; 2 Department of Obstetrics and Gynecology, Faculty of Medicine, University of Malaya, Kuala lumpur, Malaysia

**Keywords:** Environmental tobacco smoke, Stress, Miscarriage, Preterm births

## Abstract

**Back ground:**

Exposure of pregnant women to environmental tobacco smoke has been shown to be associated with low birth weight. Many studies have suggested that stress have a role in the etiology of preterm birth.

**Aims:**

This study carried out from June 2008 to March 2009 to find the relation between environmental tobacco smoke, stress and miscarriage and preterm births.

**Methods:**

A total of 33 subjects consisted of multiparous pregnant women that were in their early third trimester were chosen for this investigation. Subjects were divided into test group women with adverse pregnancy outcome, control group women with successful pregnancy. Four ml of unstimulated whole saliva were collected. The concentrations of cotinine and cortisol were evaluated using commercially available ELISA kit.

**Results:**

Pregnancies in which the average standardized cortisol during history of previous miscarriage(s) which occurred within 6th–27th week or/and history of preterm labor which occurred within 28th–36th weeks of gestation, demonstrated higher cortisol level (1.0201 ± 0.1855 ng/ml) compared to control group 0.9757 ± 0.2860 ng/ml (*P* = 0.323); statistical analysis showed no significant differences. Women of control group were more likely to be environmental tobacco smoke exposed (1.2714 ± 1.7639 ng/ml) than women with miscarriage and preterm births (0.9889 ± 0.5498 ng/ml).

**Conclusion:**

The results from this primarily study demonstrated no association between cotinine, cortisol, miscarriage and preterm births.

## Introduction

There is growing awareness of the harmful effects of smoking during pregnancy. Smoking, through exposure to substances like nicotine and carbon monoxide, is associated with a number of serious complications during pregnancy, including increased rates of spontaneous abortion [1], premature delivery [1, 2] and low birth weight [1]. According to WHO/ICD, miscarriage is defined as the death of fetus before 22 weeks of gestation or a fetus delivered weighing less than 500 g, so the preterm birth will include delivery after 22 weeks and before 37 weeks of gestation.

Exposure of pregnant women to environmental tobacco smoke has been shown to be associated with low birth weight in the infants of exposed non-smoking mothers [2, 3], and to have an added negative effect on the infant’s birth weight in smoking mothers [2]. Smoking during pregnancy also leads to an increased infant death rate around the time of birth (perinatal mortality) [4], up to one and a half times the average rate [2]. Studies of environmental tobacco smoke (ETS) and risk of spontaneous abortion are limited to a few studies of self-reported exposure to ETS or paternal smoke, and the results have been inconsistent [5–7]. Studies based on self-reported ETS exposure probably provide even larger validity concerns than self-reported active smoking and do not properly account for all possible exposures at home, work, and in public places [8, 9]. Measurement of cotinine, a biomarker of nicotine, has been shown to be a valid summary measure of the dose received from ETS as well as from active smoking [8–10].

Cortisol is commonly used as a stress marker because its production by the adrenal cortex tends to increase as a result of energetic, immunological, and psychological challenges [11, 12]. There is growing evidence that psychological, social and economic stresses increase risks of preterm birth and prenatal mortality. It is postulated that stress-induced elevations in cortisol and catecholamine alter the immune response and increase free placental corticotrophin-releasing hormone (CRH) that may act as an uterotonic agent [13]. Many studies have suggested that stress and stress hormones have a role in the etiology of preterm birth (PTB) [11–13].

Oral fluid has attracted widespread interest as a diagnostic medium for rapid, point-of-care testing [14–16]. Saliva has been considered a “mirror of the body” that generally reflects the state of a patient’s overall health. There is growing interest in the use of human whole saliva for diagnostics and disease monitoring as an alternative to blood samples. In contrast to blood, whole saliva is a non-sterile body fluid. Proper handling and storage are required to preserve the integrity of potential biomarkers [17]. The advantages of using saliva for disease diagnostics include ease of access, noninvasive sample collection, increased acceptance by patients, and reduced risks of infectious disease transmission [16]. Oral samples are readily accessible as whole saliva or by sampling secretions from specific glands. The aim of the study was to evaluate the association between ETS and stress as assessed by salivary cotinine and cortisol levels with miscarriage and preterm births.

## Materials and methods

### Sample population

This was a cross-sectional study carried out in Malaysia, Kuala Lumpur over 9 months, from June 2008 to March 2009. A total of 250 pregnant women who had their antenatal check-up at the Antenatal Clinic, University Malaya Medical Centre (UMMC) were examined prior to being referred to The Periodontal Postgraduate Clinic, Faculty of Dentistry, University of Malaya for saliva collection. Sixty-three subjects who fulfilled a set of inclusion and exclusion criteria were only eligible for this study.

Thirty-three volunteered pregnant women agreed to participate in this study. Women gave informed consent for all aspects of the study, which was approved by the Ethical Committee. The study protocol was approved by The Medical Ethics Committee of UMMC (No: 607.1) and The Ethical Committee Board of the Faculty of Dentistry, University of Malaya (No: DF OP0706/0029 (P), a).

### Medical records

The medical records of all subjects were gathered from the Antenatal Clinic of UMMC. The information collected included history of previous pregnancy, expected date of delivery (EDD), patient’s medical status and presence of gestational diabetes in current pregnancy. The presence of serious medical or systemic condition will exclude sample participation in the study.

Based on the information in the medical records, subjects were divided into two groups:

1. *Test group*: Women with miscarriage and preterm births, 18 of healthy pregnant women who had history of previous miscarriage(s) which occurred within 6th–27th week or/and history of preterm labor which occurred within 28th–36th week of gestation.
2. *Control group*: Women with successful pregnancy, 14 healthy pregnant women who had no previous miscarriage or preterm delivery at more than 36 weeks.

### Inclusion and exclusion criteria

Thirty-three pregnant women registered at the Antenatal Clinic, UMMC aged between 23- and 39-years old were selected for the study. The inclusion criteria were gestational age between 24 and 32 weeks and multiparous. The exclusion criteria were: smoking active or passive at work or at home, drug abuse such as prednisone, dexamethasone, medical or systemic conditions such as cardiovascular disease, respiratory problem, diabetes mellitus, kidney disease, hematological disorder and other chronic diseases, gestational diabetes and multiple foetuses.

The selected subjects were asked to fill a questionnaire which included information on age, ethnicity, EDD, current body weight, gestational weight gain, occupation, and pregnancy history. Subjects were asked to spit into sterile plastic tubes to collect 4 ml of unstimulated whole saliva which was then kept frozen at −80 °C until analyzed. The collection of the saliva was standardized before 12:00 p.m. to avoid differences caused by circadian rhythm.

### Enzyme-Linked Immunosorbent Assay Technique (ELISA)

The concentrations of cotinine and cortisol from the saliva samples were evaluated using commercially available ELISA kit **(**NANO LIFE QUEST SDN. BHD. SIGMA, GERMANY). ELISA technique is a biochemical procedure to detect the presence of an antibody or antigen from any sample such as plasma, vaginal fluid or saliva. Basically, this technique involves an affixation of an antigen of unknown amount to a microwell surface which has been pre-coated with an antibody specific for the antigen in question.

### Statistical analysis

Statistical Package for Social Science (SPSS) Version 16.0 was used to analyze the results. Since the cotinine and cortisol levels in the saliva displayed a non-normal distribution, the Mann–Whitney test was used to determine the significance of the difference between the test and control groups, for which the statistical significance was set for a *p* value of ≤0.05.

## Results

### Sample population

A total of 33 subjects consisted of multiparous pregnant married Malaysian women (Malayan ethnicity) that were in their early third trimester were chosen for this investigation. The age range of the subjects was between 23- and 39-years old with a mean age of 29.8 (±3.6) years. The mean age for the test group was 29.3 and for control group was 30.7. All subjects were Malay government employers. Women with miscarriage and preterm births and women in successful pregnancy group had approximately the same age. Table 1 showed the demographic distribution of the sample.

**Table 1:** Sample population between test and control groups

	Total sample	Test group (*n* = 20)	Control group (*n* = 13)
Age	33	29.3 ± 4.0	30.7 ± 3.0
*Race*			
Malay	33	20 (100 %)	13 (100 %)
*Marital status*	33		
i. Single		0	0
ii. Married		33 (100 %)	33 (100 %)
Gestational weight increment	33		
i. <5 kg		1	2
ii. 6–10 kg		7	6
iii. 10–20 kg		3	4
iv. 20–30 kg		4	0
v. >30 kg		0	1
Gestational age upon examination	33	29 (±3.0) weeks	26.82 (±6.2) weeks

**Fig. 1:**
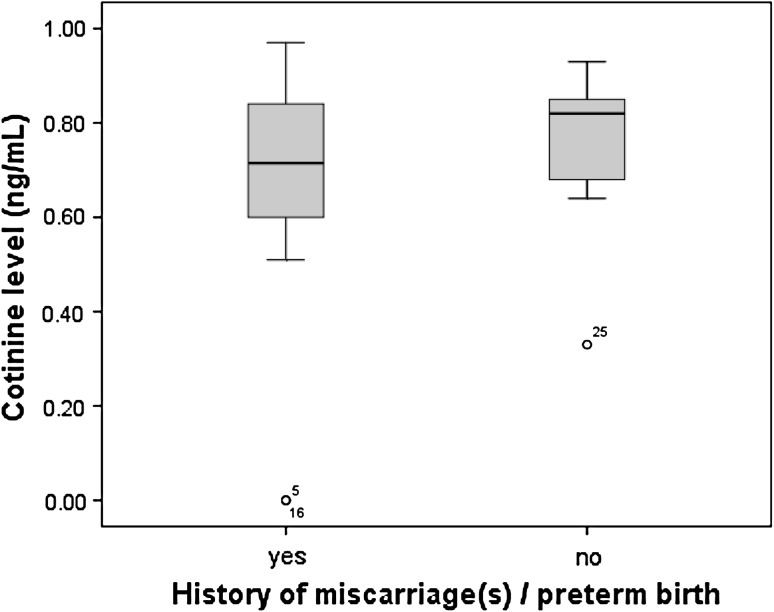
Level of cotinine verses history of miscarriage and preterm births

Results demonstrated that saliva cotinine level was higher in control group (0.7485 ± SEM 0.1589 μg/ml) compared with test group (0.6776 ± SEM 0.2658 μg/ml). Women control group were more likely to be ETS-exposed than women with miscarriage and preterm births. statistical analysis showed no significant differences (Table 2). Figure 1 demonstrates the comparative history of miscarriage and preterm births according to ETS-exposed by evaluating cotinine level in both groups. There were two outliers in test group and one outlier in control group. The level of cotinine for these three outliers is very much low compared to other subjects in each group. Results also show no significant differences.

**Table 2:** Cotinine level in test and control groups

Group	*n*	Mean ± SEM (μg/ml)	Median (μg/ml)	*p* value
Test	20	0.6776 ± 0.2658	0.7150	0.598*
Control	13	0.748 5 ± 0.1589	0.8200	

* Mann–Whitney Test

**Fig. 2:**
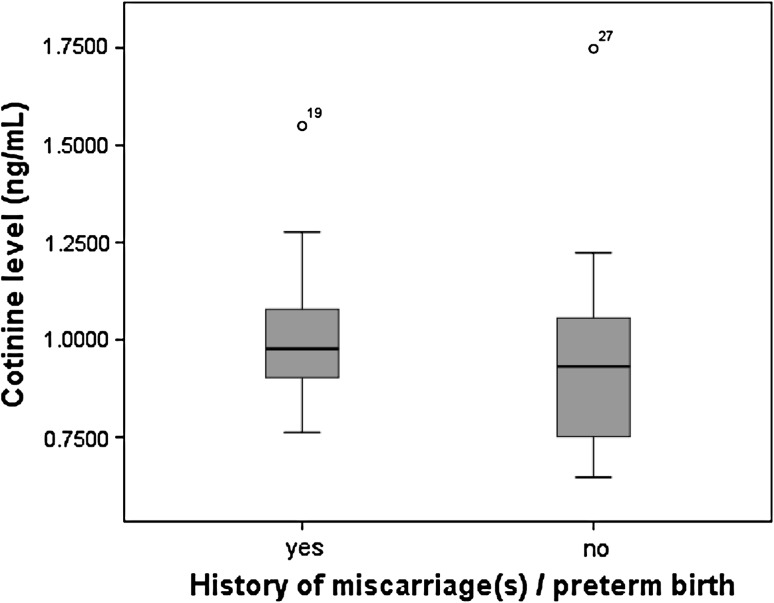
Level of cortisol verses history of miscarriage and preterm births

We calculated the comparative history of miscarriage and preterm births according to cortisol exposure. Pregnancies in which the average standardized cortisol during history of previous miscarriage(s) which occurred within 6th–27th week or/and history of preterm labor which occurred within 28th–36th weeks of gestation, demonstrated higher cortisol level (1.016 ± SEM 0.182 μg/ml) compared to control group 0.978 ± SEM 0.298 μg/ml (*P* = 0.392), normal morning levels of saliva cortisol is 0.99 ± 0.42 μg/100 ml, however, statistical analysis showed no significant differences. [Table 3, Fig. 2]. As for the cotinine, there were also outliers in each group. However, the levels of cortisol for these outliers were higher compared to other subjects in the groups.

**Table 3:** Cortisol level in test and control groups

Group	*n*	Mean ± SEM (μg/ml)	Median (μg/ml)	*p* value
Test	20	1.016 ± 0.182	0.977	0.392*
Control	13	0.978 ± 0.298	0.932	

* Mann–Whitney Test

## Discussion

To our knowledge, there are a few studies on the relation between ETS and stress as risk for miscarriage and preterm births based on cotinine and cortisol measurements. In recent years, there has been growing knowledge and awareness of the dangers and serious adverse health effects of environmental tobacco smoke exposure. Non-smokers hotel workers in London show saliva cotinine level (0.5 ng/ml) [18]. The health hazards of environmental tobacco smoke exposure affect almost every organ and system in the body with a wide spectrum of ailments and diseases, and it has been clearly implicated as the cause of death in many of those who were exposed to it [19]. Environmental tobacco smoke exposure acquires special importance when it comes to considering the negative health impact on children.

There is much evidence to show that smoking complicates the course of pregnancy, endangers the life of the mother, threatens the life of the fetus, and places the newborn at great risk of immediate and long-term complications, and possibly even death [20].

We found that women with successful pregnancy and women with miscarriage and preterm births were exposed to ETS. We decided a priori to exclude smoking women and women using oral snuff or nicotine replacement therapy. Our finding showed that women with successful pregnancy outcome represent higher level of cotinine in their saliva samples. Statistical analysis could not show a significant differential effect of exposure to ETS. This may because the power to study miscarriage and preterm births was limited, because our sample included only 13 cases with normal and 20 with abnormal pregnancy. The biologic mechanisms underlying a possible association between ETS and miscarriage and preterm births may involve pathways similar to those for active smoking, because side stream tobacco smoke contains many of the same constituents as mainstream tobacco smoke [18]. Several components of tobacco smoke (e.g, nicotine, carbon monoxide, and cyanide) are toxic for the developing fetus. Nicotine has vasoconstrictive effects leading to reduced placental blood flow [19]. Carbon monoxide binds to hemoglobin, causing maternal and fetal hypoxia, which may interfere with the development of the growing fetus and induce fetal death. Research indicates that later in pregnancy, smoking does appear to decrease the placenta’s ability to deliver nutrients to the developing baby. In addition to potentially causing miscarriages, this can cause babies to be born with lower birth weight and can also increase the risk of stillbirth, as well as death in the first year of life [18, 20].

Researchers found that 90 percent of women, whose ages ranged from 18 to 34, with elevated levels of the stress-induced hormone miscarriage during the first 3 weeks of pregnancy, compared to 33 percent of those with normal levels. Researchers suggest that the body may recognize elevated cortisol levels as an alarm that conditions are unfavorable for pregnancy [21, 22].

Maternal stress is commonly cited as a potential cause for at least part of pregnancy losses that remain “unexplained” [21–23]. Yet, for humans, little physiological evidence exists in support of this hypothesis [24, 25].

Our finding of an association between increased maternal cortisol and higher risk of miscarriage and preterm births, together with previous research has failed to find such an association [25]. Cortisol secretion can be affected by circadian rhythms, physical activity, food consumption, smoking, caffeine, alcohol, and steroid medications [26–28]. None of our participants smoked or consumed alcohol. Cortisol levels may be affected by age [29]; the lack of an effect of age on cortisol may be due to the youthfulness of our sample. The 32 women who conceived during the study ranged from 23 to 39 years, but the age distribution was heavily weighted toward the early twenties.

## Conclusion

Results demonstrated no association between cotinine, cortisol miscarriage and preterm births. Future longitudinal studies with larger samples will be necessary to compare cortisol and cotinine levels as risk factors for miscarriage and preterm births across the entire duration of gestation. Further research will also be necessary to explore the physiological pathways that might mediate the observed association.
